# A Putative Receptor for Ferritin in Mollusks: Characterization of the Insulin-like Growth Factor Type 1 Receptor

**DOI:** 10.3390/ijms24076175

**Published:** 2023-03-24

**Authors:** Bowen Huang, Qin Liu, Changming Bai, Chen Li, Chongming Wang, Lusheng Xin

**Affiliations:** 1 Function Laboratory for Marine Fisheries Science and Food Production Processes, Qingdao National Laboratory for Marine Science and Technology, Yellow Sea Fisheries Research Institute, Chinese Academy of Fishery Sciences, Qingdao 266071, China; 2 Guangxi Key Lab of Agricultural Resources Chemistry and Biotechnology, Yulin Normal University, Yulin 537000, China

**Keywords:** insulin-like growth factor type 1 receptor, ferritin, iron metabolism, fibronectin type III domain, *Scapharca broughtonii*

## Abstract

The ferritin secreted by mammals has been well documented, with the protein capable of localizing to cell membranes and facilitating the delivery of iron to cells through endocytosis. However, the presence of ferritin in the circulatory fluid of mollusks and its functions remain largely unknown. In this study, we aimed to investigate the potential interacting proteins of ferritin in the ark clam (*Sb*Fn) through the use of a pull-down assay. Our findings revealed the presence of an insulin-like growth factor type 1 receptor (IGF-1R) in ark clams, which was capable of binding to *Sb*Fn and was named *Sb*IGF-1R. *Sb*IGF-1R was found to be composed of two leucine-rich repeat domains (L domain), a cysteine-rich domain, three fibronectin type III domains, a transmembrane domain, and a tyrosine kinase domain. The ectodomain of *Sb*IGF-1R was observed to form a symmetrical antiparallel homodimer in the shape of the letter ‘A’, with the fibronectin type III domains serving as its ‘legs’. The mRNA expression of *Sb*IGF-1R gene was detected ubiquitously in various tissues of the ark clam, with the highest expression levels found in hemocytes, as determined by qRT-PCR. Using a confocal microscopic and yeast two-hybrid assays, the interaction between *Sb*IGF-1R and *Sb*Fn was further verified. The results showed that *Sb*Fn co-localized with *Sb*IGF-1R on the cell membrane, and their interaction was expected to occur on the FNIII domains of the *Sb*IGF-1R. In conclusion, our findings highlight the identification of a putative receptor, *Sb*IGF-1R, for *Sb*Fn, demonstrating the versatility of IGF-1R in ark clams.

## 1. Introduction

Iron is an essential catalytic cofactor of numerous enzymatic reactions that participate in fundamental cellular metabolic processes, such as energy generation, protein synthesis, and nucleic acid synthesis [1,2]. Nevertheless, the intrinsic redox properties of iron promote its involvement in the production of reactive oxygen species, which can lead to oxidative damage to cellular macromolecules, including membrane lipids, that disrupts cellular homeostasis and can contribute to pathogenesis [3]. Vertebrates have developed several mechanisms to modulate iron homeostasis, including uptake, transport, storage, and utilization of iron, to maintain the balance between the necessary biological functions of iron and its harmful effects. Among these regulatory mechanisms, ferritin is a central player that regulates iron levels by storing iron within cells and preventing the production of harmful reactive oxygen species [4,5,6].

Ferritin features a nanocage that can store roughly 4500 iron atoms in a bioavailable state that serves as a buffer for reactive oxygen species inside cells [7]. In vertebrates, ferritin also operates as a non-transferrin iron transport mechanism in circulation, managing the distribution of iron homeostasis across the whole organism [8,9]. Several cell surface endocytosis receptors for vertebrate ferritin have been identified [8,10,11]. Specifically, murine T-cell immunoglobulin and mucin domain protein 2 (TIM2) were found binding to H-ferritin and internalizing it into endosomes and lysosomes [11]. The transferrin receptor (TfR) was subsequently identified as an endocytic receptor for H-ferritin that mediates human lymphocytes and reticulocytes interaction with ferritin [8]. Thereafter, scavenger receptor class A member 5 (SCARA5) was shown to play a role in ferritin uptake by binding to L-ferritin and then delivering iron through endocytosis [10].

As a highly conserved iron storage molecule in nature, ferritin exists in a variety of species, from mammals to archaea [12]. The levels of ferritin in the hemolymph of invertebrates have been shown to be roughly 1000-fold greater than the ferritin levels in vertebrate plasma [13,14,15,16]. *Drosophila melanogaster*’s secretory ferritin transports iron across the basolateral membrane of enterocytes, contributing to dietary iron delivery [14]. Accumulation of ferritin in the marine mollusk ark clam, *Scapharca broughtonii*, was discovered to mirror iron distribution [17,18]. Considering the highly conserved structure-function features of invertebrate ferritin, a ferritin iron delivery mechanism akin to that of vertebrates undoubtedly works in invertebrates, although no ferritin receptors in invertebrates have been reported until now [19,20].

The insulin-like growth factor (IGF) system plays a crucial role in the growth, reproduction, and development of aquatic animals, and has been identified as a useful biomarker for evaluating intra-species growth differences in aquaculture [21,22]. Research on this system has been conducted in various bivalves, including the hen clam *Mactra heinesis*, surf clam *Spisula sachalinensis*, yesso scallop *Patinopecten yessoensis*, pacific oyster *Crassostrea gigas*, and mussel *Mytilus galloprovincialis* [23,24,25,26,27]. The insulin-like growth factor type 1 receptor (IGF-1R) is a homodimer receptor tyrosine kinase with a transmembrane tetrameric structure. Each IGF-1R α-β pro-receptor consists of a receptor L domain-1, cysteine-rich domain, a receptor L domain-2, a fibronectin type III domain-1, -2, -3, transmembrane domain, and a tyrosine kinase domain [28]. It is cleaved by proteolysis and glycosylation into functional mature-form receptors that consist of two identical extracellular α- chains and two identical β-chains, with the chains linked by disulfide bridges to form stable homodimers. The α chain located outside the cell membrane forms a glycosylated ligand binding domain and the β chain contains an extracellular domain, a transmembrane domain, and an intracellular tyrosine kinase domain. IGF-1R is an essential component of the IGF system, triggering autophosphorylation upon ligand binding and internalization mediated by clathrin or caveolin-1, activating intracellular signaling cascades, such as MAP kinase and PI3-kinase, to regulate cell growth and senescence [29,30,31].

In the current study, a tyrosine kinase receptor member gene was captured as a potential interacting protein of ferritin in ark clams (*Sb*Fn) by pulldown assay, named *Sb*IGF-1R, and its mRNA expression pattern was detected. The interaction between *Sb*IGF-1R and *Sb*Fn was further verified by confocal microscopic and yeast two-hybrid assays. Finally, a putative membrane anchor, an insulin-like growth factor type 1 receptor, for ferritin in mollusks was revealed, implying an important role of ferritin in iron delivery among cells.

## 2. Results

### 2.1. Identification of Potential Membrane Anchors for SbFn

**Figure 1:**
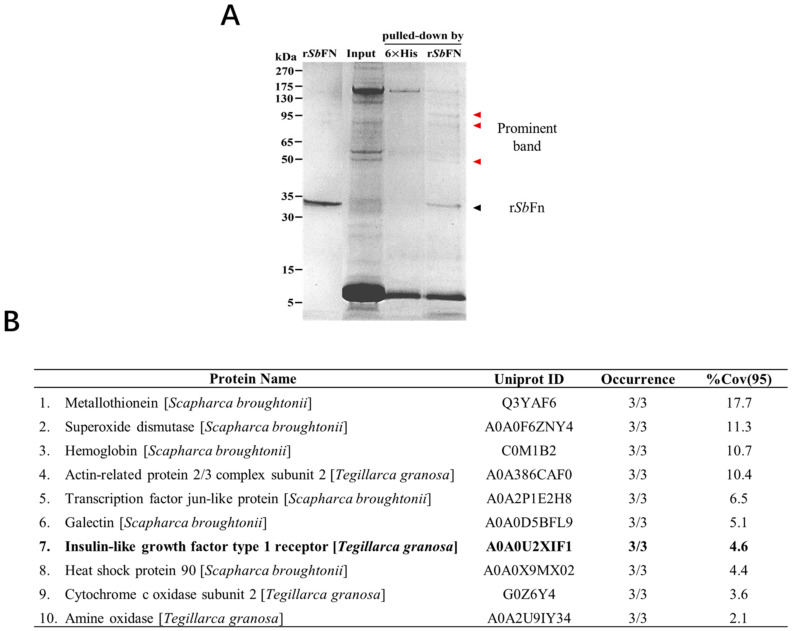
Identification of the *Sb*Fn interacting protein by His pull-down assay. (**A**) The recombinant His *Sb*Fn served as bait to capture the binding protein from hemocyte lysate. Hexa-His was employed as the control. The binding protein was directly analyzed by SDS-PAGE, and the different band is indicated by the arrow and characterized by LC-MS/MS. (**B**) Candidate proteins identified by LC-MS/MS interacting with *Sb*Fn. Occurrence refers to the times this protein was detected in three replicate experiments. %Cov (95) refers to the percentage of all identified polypeptides relative to the total amino acid sequence at 95% confidence.

To investigate the mechanism of ferritin transfer in invertebrates, we screened *Sb*Fn-interacting proteins using His-tagged *Sb*Fn as the bait in ark clam hemocytes. The obtained preys were separated by SDS-PAGE, and the His-*Sb*Fn group showed unique bands of 50, 85, and 95 kDa (Figure 1A). By using LC-MS/MS, several candidate proteins were identified, including amine oxidase, cytochrome c oxidase subunit 2, and insulin-like growth factor type 1 receptor (IGF-1R) (Figure 1B). To continue our investigation, we chose IGF-1R, a member of the receptor tyrosine kinases (RTKs), a large family of cell surface receptors.

### 2.2. Molecular Characterization of SbIGF-1R

**Figure 2:**
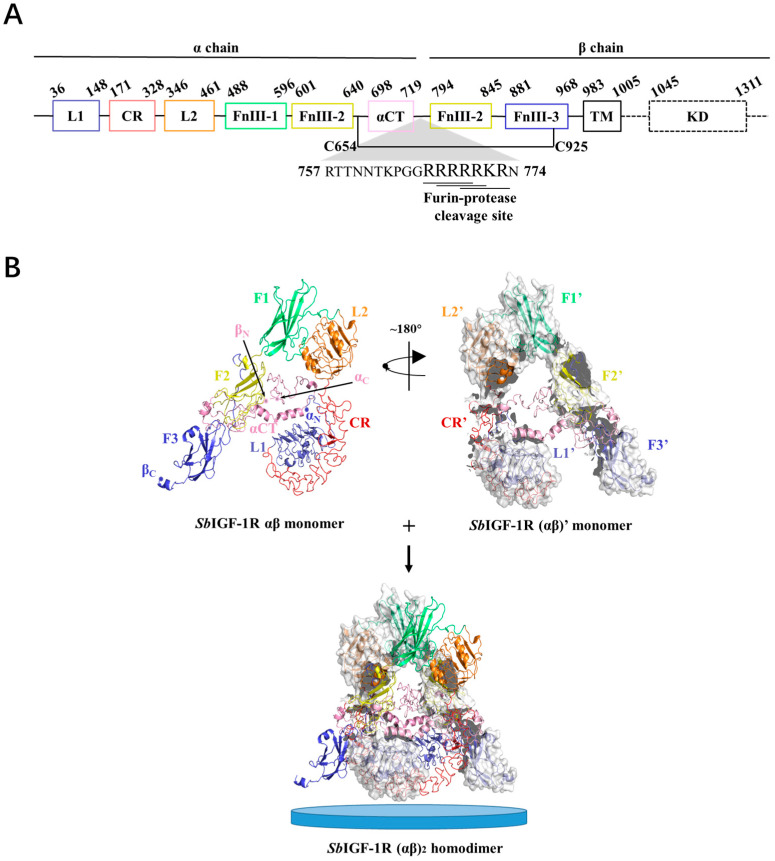
Three-dimensional structure of the *Sb*IGF-1R ectodomain. (**A**) Domain organization of *Sb*IGF-1R. (**B**) Overall view of the *Sb*IGF-1R ECD homodimer. The structure of the αβ monomer shows that it pairs with the (αβ)’ monomer to form an (αβ)2 homodimer. The N and C terminals of the α and β chains of *Sb*IGF-1R ECD are indicated as αN, αC, βN, and βC, respectively. The blue discs represent cell membranes.

A potential protein of 1475 amino acid residues was encoded by the 4425-bp open reading frame (ORF) of the full-length *Sb*IGF-1R cDNA (with a predicted molecular mass of 168.3 kDa and a theoretical pI of 6.62). The sequence is available in GenBank with the accession number OM055760. The precursor protein includes a conserved furin recognition motif RXXR located at residues 767–773, flanked by α chain and a β chain (Figure 2A). According to SMART prediction domain analysis, the *Sb*IGF-1R α chain was found to be composed of two receptor L domains (L1 and L2), a cysteine-rich domain (CR), a fibronectin type III domain-1 (F1), and a portion of fibronectin type III domain-2 (F2). A portion of F2, fibronectin type III domain-3 (F3), the transmembrane domain (TM), and the tyrosine kinase domain (KD) were found in the β chain (Figure 2A).

**Figure 3:**
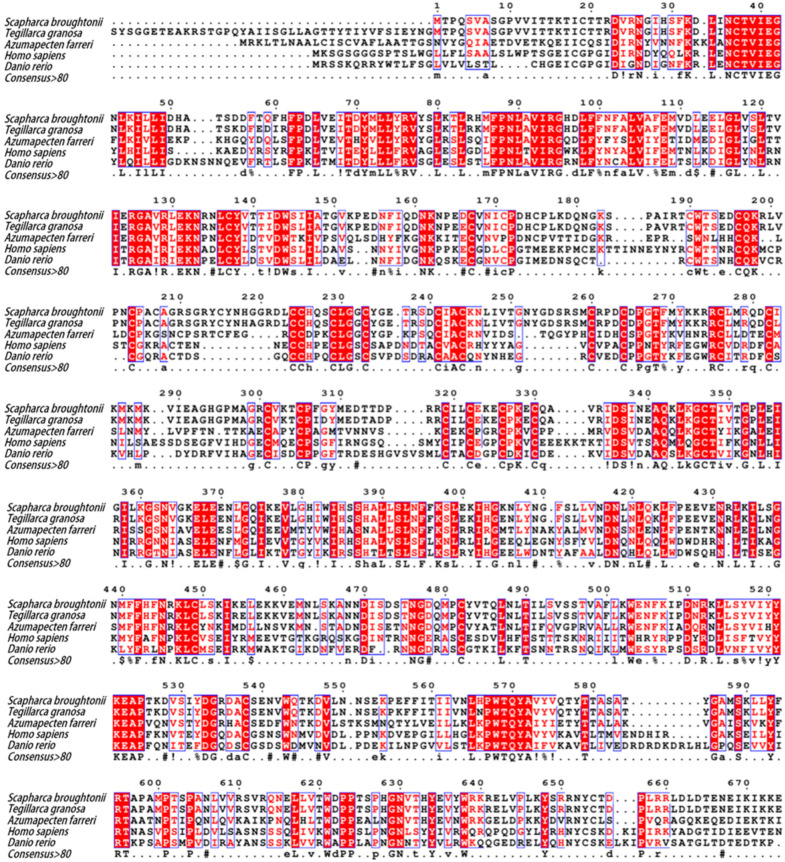

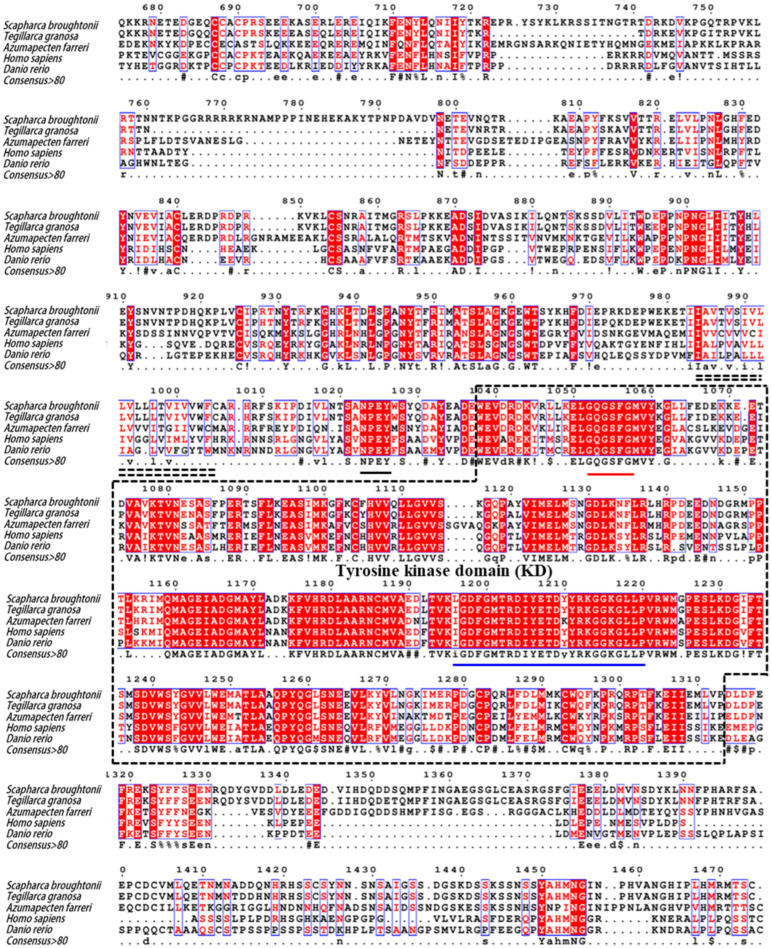
Multiple sequence alignment of *Sb*IGF-1R with other IGF-1Rs from various species. The amino acid number above the sequence refers to *Sb*IGF-1R. Identical and similar amino acid residues are indicated with minuscules. The double-dashed amino acid fragments represent the transmembrane domain. The ATP binding motif (red), the activation loop (blue), and the NPXY motif (black) are underlined in different colors. In the consensus sequence: upper case is identity, lower case is consensus level > 0.5, ! is stands for I or V, $ for L or M, % for F or Y, # for N, D, Q, E, B or Z. The GenBank accession numbers of the aligned sequences are as follows: *Tegillarca granosa* (ALS40479.1)*, Azumapecten farreri* (QFR39799.1)*, Homo sapiens* (P08069.1)*, Danio rerio* (NP_694501.1).

The tertiary structure of *Sb*IGF-1R ECD was calculated using the I-TASSER online server, which was selected from five predicted models based on the C-score (−0.63) with a TM-score of 0.63 ± 0.13 and an RMSD of 10.4 ± 4.6 Å (Figure 2B). The GalaxyRefine web service was used to refine the chosen *Sb*IGF-1R ECD tertiary structure, which showed a Ramachandran plot with favored regions of 84.7% and permitted areas of 12.6% (Figure S1). The ProSA-web and the ERRAT server were used to double-check the revised model for any flaws in quality and error incidence. The tertiary structure, as refined, has an ERRAT quality factor of 80.6554 and a ProSA-web Z-score of −9.39. ZDOCK suggests that the *Sb*IGF-1R ECD will fold into an ‘A’-shaped symmetric antiparallel homodimer with an energy of −55.0 kcal/mol (Figure 2B). The apex of this “A” is made up of interacting pairs of L2-F1 domains from opposite chains, while the “legs” are made up of linearly folded F2 and F3 domains. The KD domain of *Sb*IGF-1R is located between residues 1038 and 1313. This domain contains conserved amino acids predicted to play an important role in protein kinase activity. The catalytic region of *Sb*IGF-1R contains an ATP binding motif (GXGXXG) at positions 1052–1057. A cluster of three tyrosine residues located within the activation loop of 1197–1222, which activates the kinase by allowing ATP and substrate to enter the active site, is essential for receptor autophosphorylation. In addition, the intracellular juxta-membrane domain contains a conserved NPXY motif, which may be critical for the internalization of the receptor (Figure 3).

**Figure 4:**
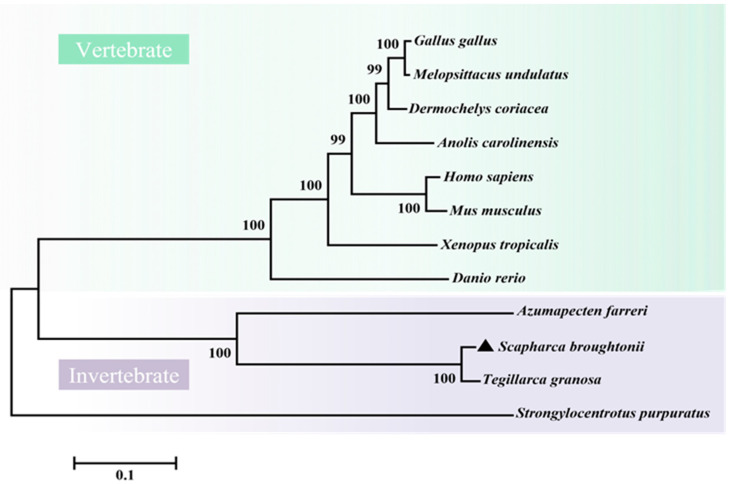
Phylogenetic tree of IGF-1Rs across species. The tree was obtained using the neighbor-joining method of MEGA 6.0. The percentage at the forks indicates the bootstrap value for 1000 replications. The black triangle indicates *Sb*IGF-1R. The accession numbers of these proteins obtained from GenBank are listed below: *Homo sapiens* IGF-1R (BAG11657.2), *Mus musculus* IGF-1R (NP_034643.2), *Danio rerio* IGF-1R (NP_694500.1), *Xenopus tropicalis* IGF-1R (XP_002933351.1), *Anolis carolinensis* IGF-1R (XP_003226539.2), *Gallus gallus* IGF-1R (NP_990363.1), *Melopsittacus undulatus* IGF-1R (XP_033922143.1), *Dermochelys coriacea* IGF-1R (XP_038274215.1), *Tegillarca granosa* IGF-1R (ALS40479.1), *Azumapecten farreri* IGF-1R (QFR39799.1), *Strongylocentrotus purpuratus* IGF-1R (XP_030832338.1).

*Sb*IGF-1R revealed 92.94% similarity to IGF-1R from *Tegillarca granosa* (ALS40479.1), 52.97% to *Azumapecten farreri* (QFR39799.1), 42.19% to *Homo sapiens* (P08069.1), and 39.58% to *Danio rerio* (NP_694501.1) (Figure 3). The IGF-1R phylogenetic tree has two primary branches: one produced by vertebrate IGF-1Rs and the other by invertebrate IGF-1Rs (Figure 4). As a result, *Sb*IGF-1R is more closely related to IGF-1R proteins found in invertebrates than to those found in higher organisms.

### 2.3. The mRNA Expression Pattern of SbIGF-1R in Various Tissues

**Figure 5:**
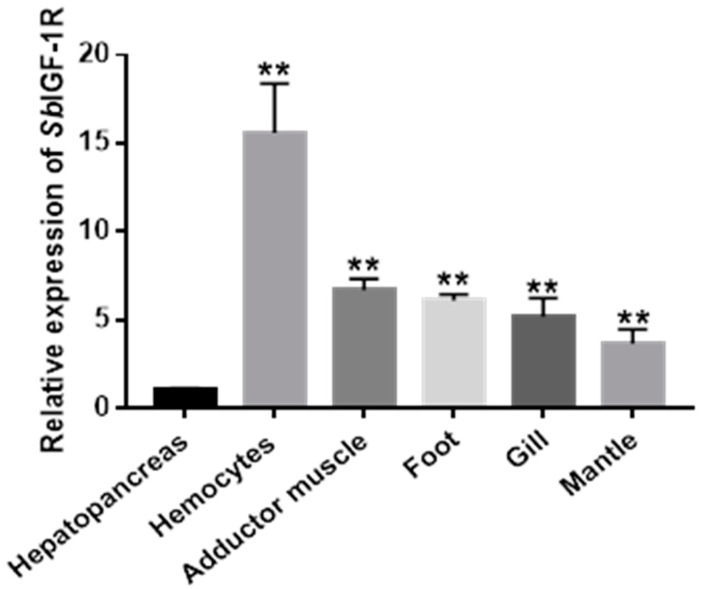
Relative mRNA expression of *Sb*IGF-1R in healthy ark clams’ tissues detected by qRT-PCR. The transcript levels in the hepatopancreas, hemocytes, adductor muscle, foot, gill, and mantle were normalized to that in the hepatopancreas. The asterisks indicate significant differences: ** *p* < 0.01.

The mRNA of *Sb*IGF-1R gene was shown to be constitutively expressed with varying degrees throughout a wide range of tissues by qRT-PCR assay, including the hepatopancreas, hemocytes, adductor muscle, foot, gills, and mantle. The hemocytes had the greatest mRNA expression level (15.59 ± 1.49-fold, *p* < 0.01), followed by the adductor muscle (6.68 ± 0.63-fold, *p* < 0.01), the foot (6.10 ± 0.36-fold, *p* < 0.01), and the gill (5.21 ± 1.02-fold, *p* < 0.01) (Figure 5).

### 2.4. The Interaction Mode between SbIGF-1R and SbFn

**Figure 6:**
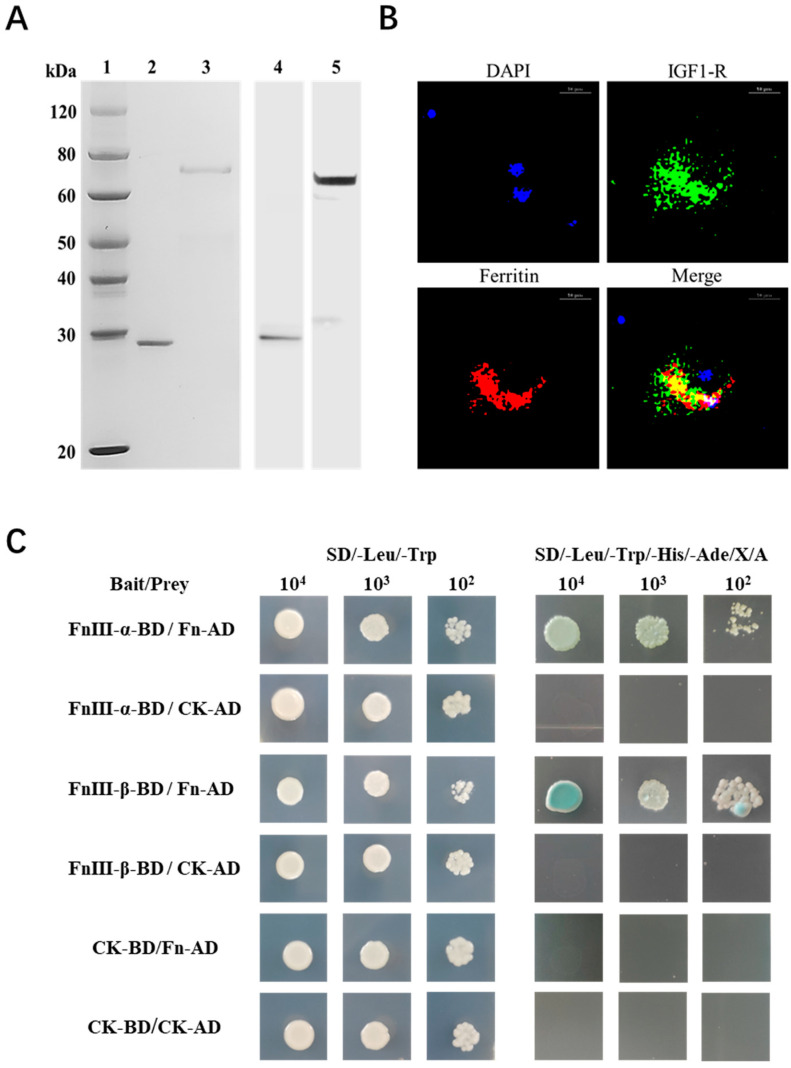
Interaction between *Sb*IGF-1R and *Sb*Fn. (**A**) Recombinant proteins of *Sb*Fn and *Sb*IGF-1R-FNIII and the specificity of their polyclonal antibodies. Line 1: Protein marker. Lines 2 and 3: Purified recombinant proteins of *Sb*Fn and *Sb*IGF-1R-FNIII, respectively. In lines 4 and 5, a Western blot analysis was performed using *Sb*Fn and *Sb*IGF-1R-FNIII polyclonal antibodies against the corresponding purified proteins. (**B**) *Sb*Fn colocalized with *Sb*IGF-1R in ark clam hemocytes. Extracellular *Sb*Fns were co-localized with *Sb*IGF-1Rs on the surface of the hemocytes. The positive *Sb*IGF-1R and *Sb*Fn signals are indicated in green and red, respectively. The nucleus is indicated by the blue signal of DAPI (scale bar = 10 μm). (**C**) Yeast two-hybrid pairwise analysis showing the interaction of *Sb*IGF-1R and *Sb*Fn. FnIII-(α/β)-BD/Fn-AD are the experimental groups; CK-BD/CK-AD is the negative control; FnIII-(α/β)-BD/CK-AD and CK-BD/Fn-AD are the self-activation testing groups.

The interaction mode between *Sb*Fn and *Sb*IGF-1R was further explored by confocal microscopic and yeast two-hybrid assays. Polyclonal antibodies against the *Sb*Fn and *Sb*IGF-1R-FNIII were prepared, and their specificity was identified by Western blot (Figure 6A). Extracellular *Sb*Fns (red) were co-localized with *Sb*IGF-1Rs (green) on the surface of the ark clam hemocytes (Figure 6B). In the yeast two-hybrid assays, FnIII-α/β-BD and Fn-AD carried yeasts were grown in SD-Leu-Trp-His-Ade/X-a-gal/Aba medium and showed a blue color (Figure 6C), indicating a potential direct interaction between *Sb*IGF-1R-FNIII and *Sb*Fn. In the self-activation testing groups, FnIII-α/β-BD and CK-AD, as well as Fn-AD and CK-BD carried yeasts grew in SD-Leu-Trp, but not in SD-Leu-Trp-His-Ade/X-a-gal/Aba selective medium (Figure 6C), indicating that FnIII-α/β and Fn could not activate the reporter genes by themselves. Neither of the negative control CK-BD/CK-AD carried yeasts grew in SD-Leu-Trp-His-Ade/X-a-gal/Aba medium (Figure 6C).

## 3. Discussion

Ferritin is a widely conserved iron-storage protein found in various species [32]. Its receptors facilitate the endocytosis of extracellular ferritins and play a vital role in the maintenance of iron homeostasis in mammals [33,34]. In our earlier investigation, we discovered secreted ferritin in the hemolymph of *S. broughtonii*; however, whether it could return to cells was unknown. In this work, we showed for the first time that the highly expressed hemocyte protein *Sb*IGF-1R could bind with *Sb*Fn, providing an anchoring site for ferritin on the cell surface.

As a receptor tyrosine kinase, IGF-1R plays a crucial role in cell-environment communication, whereby ligand binding can induce its internalization through clathrin- or caveolin-1-dependent mechanisms [28,31]. In the case of *Sb*IGF-1R, its ECD chain forms a symmetric antiparallel dimer resembling mammalian IGF-1R [35], and its fibronectin type III domain serves as a scaffold for ligand binding. *Sb*Fn directly interacted with the fibronectin type III domains of *Sb*IGF-1R, as shown by the yeast two-hybrid experiment. Expectedly, the fibronectin type III domain interacts with *Sb*Fn. This domain is an autonomously folded unit that exists in numerous receptors and serves as a scaffold for ligand binding [36]. As is well-known, the fibronectin type III domains participate in the binding of gliadin, the interleukin 6 receptor, the interferon receptor 1, and the insulin-like growth factor receptor to their ligands [37,38,39,40]. Moreover, the *Sb*IGF-1R juxta-membrane domain contained a conserved NPXY motif that was supposed to recruit the clathrin adaptor, such as disabled-2, ARH, and NUMB, as a sorting signal for endocytosis [41,42], in turn binding to AP-2 or clathrin to induce clathrin-mediated cargo endocytosis [43,44], indicating that *Sb*IGF-1R might media *Sb*Fn internalization similarly.

The hepatopancreas and hemocytes are two of the major Iron-storing organs in molluscans. The iron content of the hepatopancreas and hemocytes of healthy ark clams was significantly higher than other tissues and similar to that of the freshwater mussels, *Elliptio complanata*, previously tested [17,45]. Hemocytes may play a significant role in the recycling of ferritin-bound iron ions, and the mRNA level of the *Sb*IGF-1R gene was found to be significantly expressed in the hemocytes of ark clams. Resembling macrophages in larger vertebrates, the hemocytes of ark clams were supposed to recycle extracellular iron ions [46]. For one thing, this recycling process is crucial to iron homeostasis and the redistribution of iron ions throughout the body. For another, it may reduce the infection caused by pathogens by limiting the pathogen’s access to host iron ions. Here, hemocytes with highly expressed *Sb*IGF-1R were supposed to participate in the molluscan immune defense system by iron-withholding. As we have previously reported that ferritin-bounded iron ions would be re-distributed in response to an OsHV-1 infection [17], *Sb*IGF-1R might conduct the iron accumulation in the hemocytes after OsHV-1. Further investigation is needed to explore more details of iron re-distribution after OsHV-1 infection.

In conclusion, a full-length cDNA of the IGF-1R was identified and characterized in the mollusk *S.broughtonii*. *Sb*IGF-1R exhibits a high degree of sequential and structural conservation with other IGF-1Rs and is most highly expressed in hemocytes. The interaction between *Sb*IGF-1R and *Sb*Fn was verified by confocal microscopic and yeast two-hybrid assays. These findings suggest that *Sb*IGF-1R is a potential endocytic receptor for *Sb*Fn and may provide significant insight into the mechanisms of iron metabolism in molluscans.

## 4. Materials and Methods

### 4.1. Animals and Sample Collection

Healthy adult ark clams (50 ± 12 mm in length) were obtained from a local farm in Yantai, China. Before treatment, these ark clams were acclimatized in aerated seawater at 18 °C for 1 week. The hemocytes from the ark clams were collected and centrifuged at 800× *g* for 5 min for RNA preparation and protein extraction. Meanwhile, other tissues, including the gill, mantle, hepatopancreas, adductor muscle, and foot were also collected for RNA extraction.

### 4.2. His-Fusion Proteins and His Pull-Down Assays

The sequence encoding *S.broughtonii* ferritin (*Sb*Fn, KP123597.1) was amplified by PCR with the primers listed in Table 1. The amplified fragments were digested with *Bam*HI and *Xho*I and ligated into the pET-30 (a) + vector (Novagen, Madison, WI, USA). The His-fusion protein was overproduced in the *Escherichia coli* Transetta (DE3) (TransGen, Beijing, China) under the induction of 0.5 mM isopropyl β-D-1-thiogalactopyranoside (IPTG) at 37 °C for 6 h, then was purified using Ni-Charged Resin (GenScript, Nanjing, China) according to the manufacturer’s protocol. To remove excess salt ions, the purified protein was dialyzed three times in PBS and concentrated using Millipore ultrafiltration tubes.

A pull-down assay was performed using the Pierce^TM^ His Protein Interaction Kit (Thermo Fisher Scientific, Waltham, MA, USA) to identify the *Sb*Fn-interacting proteins. The input hemocyte proteins were prepared with a cell lysis buffer supplemented with EDTA-free protease inhibitors (Roche, Basel, Switzerland). HisPur Cobalt Resin was incubated with recombinant *Sb*Fn (r*Sb*Fn) and an equal amount of Hexa-His (GenScript), respectively, for 1 h at 4 °C with gentle rocking. After 5 washes with a wash solution containing 10 mM imidazole (GenScript), the prepared r*Sb*Fn-conjugated beads were incubated with the hemocyte proteins at 4 °C overnight with gentle rocking. Meanwhile, Hexa-His-conjugated beads were incubated with an equal volume of cell lysate as a negative control. These beads were collected by centrifugation after 5 subsequent washes. The bound proteins were finally eluted with a wash solution containing 300 mM imidazole (GenScript) and separated by gradient SDS-PAGE gels at concentrations of 8–16% (GenScript). After staining with Coomassie blue, the visual intensities of the differential protein bands between the experimental and control groups were analyzed using Image J software, and bands with ≥2-fold higher abundance (*p* < 0.05) relative to the control group were excised for liquid chromatography-tandem mass spectrometry (LC-MS/MS) analysis. The LC-MS/MS analysis was performed at Guangzhou Gene Denovo Biotechnology Co., Ltd. (Guangdong, China). Briefly, the excised bands were subjected to protease degradation. The peptide samples were then separated by nanoliter liquid chromatography and detected by mass spectrometer LTQ Orbitrap Velos (Thermo Fisher Scientific, San Jose, CA, USA).

### 4.3. Clone and Bioinformatic Analyses of SbIGF-1R

The total RNA was extracted from the hemocytes with a TRIzol reagent (Invitrogen, Waltham, MA, USA), and the first-strand cDNAs were reverse-transcribed by M-MLV reverse transcriptase (Promega, Madison, WI, USA) according to the manufacturer’s instructions. The sequence encoding the complete open reading frame of *Sb*IGF-1R was obtained from the *S. broughtonii* genome. The specific primers (Table 1) were designed to amplify the full-length cDNA of *Sb*IGF-1R. The PCR products were purified from 1.5% agarose gels using a DNA gel extraction kit (Omega, Norwalk, CT, USA), ligated into the pMD 19-T vector (Takara, Dalian, China), and transformed into *E.coli* for sequencing confirmation at Shanghai Biotech (Shanghai, China). The Expasy server (https://web.expasy.org/translate/, accessed on 20 June 2022) was used to infer the amino acid sequence. The protein-conserved domains were identified using the SMART program (http://smart.embl-heidelberg.de/, accessed on 20 June 2022) and the NCBI Conserved Domain Database (https://www.ncbi.nlm.nih.gov/Structure/cdd/wrpsb.cgi, accessed on 20 June 2022). The BLASTP program (http://www.ncbi.nlm.nih.gov/blast, accessed on 21 June 2022) was used to analyze the homolog. The Clustal 1.81 software was used to perform the sequence alignment of IGF-1Rs, and the results were generated in the online platform (https://espript.ibcp.fr/ESPript/cgi-bin/ESPript.cgi, accessed on 21 June 2022). A phylogenetic tree of IGF-1Rs was constructed based on the full-length amino acid sequences by the neighbor-joining algorithm using Mega 6.0. The tertiary structures of *Sb*IGF-1R ectodomain (ECD) were calculated using I-TASSER (https://zhanglab.ccmb.med.umich.edu/I-TASSER, accessed on 23 June 2022) and further refined by using the GalaxyRefine online server (https://galaxy.seoklab.org/cgi-bin/submit.cgi?type=REFINE, accessed on 26 June 2022). In addition, the geometric quality of the *Sb*IGF-1R ECD tertiary structure was validated based on Ramachandran plots and ERRAT quality factors from the Structural Analysis Validation Server (https://saves.mbi.ucla.edu, accessed on 26 June 2022), and Z-scores from the ProSA server (https://prosa.services.came.sbg.ac.at/prosa.php, accessed on 26 June 2022). The validated *Sb*IGF-1R ECD monomers were submitted to the template-free docking server ZDOCK (http://zdock.umassmed.edu/, accessed on 27 June 2022) to predict a homodimer model for *Sb*IGF-1R ECD. Finally, the predicted homodimer model of *Sb*IGF-1R ECD was analyzed using PISA (https://www.ebi.ac.uk/msd-srv/prot_int/pistart.html, accessed on 28 June 2022) to obtain the energy of the interaction.

### 4.4. qPCR Assay of SbIGF-1R Expression

The total RNA samples were extracted from the hemocytes, gills, mantle, hepatopancreas, adductor muscle, and foot of three ark clam individuals. A cDNA library was synthesized by ReverTra Ace qPCR RT Master Mix with gDNA Remover (TOYOBO, Osaka, Japan). A real-time PCR experiment was performed with a Bio-Rad CFX Connect RealTime system to detect the distribution of *SbIGF-1R* transcripts, with a 60S ribosomal protein subunit (RL15) as the reference gene [47]. The primers for the real-time PCR are listed in Table 1. The 2^-ΔΔCt^ method was adopted to calculate the mRNA relative expression levels [48].

### 4.5. Preparation of Polyclonal Antibody

The fragment encoding the *Sb*IGF-1R fibronectin type III domains was amplified using gene-specific primers (Table 1) and was subsequently cloned into the pET-30 (a)+ vector (Novagen, Madison, WI, USA). The recombinant plasmid was introduced into *E. coli* Transetta (DE3) (TransGen). The recombinant fibronectin type III domains of *Sb*IGF-1R protein (r*Sb*IGF-1R-FNIII) were induced by 0.2 mM IPTG for 6 h at 37 °C and purified using Ni-Charged Resin (GenScript). A mixture containing 100 μg r*Sb*IGF-1R-FNIII was used to immunize 4-week-old mice for the polyclonal antibody preparation, while the *Sb*Fn antiserum was prepared by immunizing a 6-month-old rabbit.

### 4.6. Western Blotting

The specificity of the *Sb*IGF-1R-FNIII and *Sb*Fn polyclonal antibodies were identified using Western blot. The purified protein samples were separated using 12% SDS-PAGE and transferred onto nitrocellulose (NC) membranes. After blocking with 5% skimmed milk powder, the membranes were incubated overnight at 4 °C with *Sb*Fn polyclonal antibody (1:500) or *Sb*IGF-1R-FNIII (1:500) polyclonal antibody. The membranes were then incubated with HRP-conjugated goat anti-rabbit IgG and goat anti-mouse IgG secondary antibodies (1:5000, Abclonal, Wuhan, China) for 1 h at room temperature with shaking. Between each step, the NC membranes were washed thrice for 5 min with TBST. Protein bands were visualized in an automated chemiluminescent gel imaging system (ImageQuant LAS 4000; GE Healthcare) using Western lighting ECL substrate (Thermo Fisher Scientific, Waltham, MA, USA).

### 4.7. Co-Locaalization Assay

Hemocytes were sampled from healthy ark clams and then deposited on an attached slide for 1 h. After washing with sterile seawater to remove unadhered hemocytes, *Sb*Fn (100 μg/mL) was added and incubated for 2 h at 18 °C. Following two washes with sterile seawater, the hemocytes were fixed with 4 % paraformaldehyde for 30 min and blocked with 5% bovine serum albumin for 30 min at room temperature. Then the hemocytes were incubated with rabbit anti-*Sb*Fn polyclonal antibodies and mouse anti-*Sb*IGF-1R-FNIII polyclonal antibodies at 4 °C overnight. After thoroughly washing, the hemocytes went through another 2 h incubation with Cy3-coupled goat anti-rabbit IgG (H + L) (AS007) and FITC-coupled goat anti-mouse IgG (H + L) (AS001) antibodies (ABclone). Finally, fluorescence was observed with DAPI staining to visualize the nuclei under a Nikon Eclipse Ti confocal microscope (Nikon Instruments Inc., Melville, NY, USA).

### 4.8. Yeast Two-Hybrid Assay

To perform the yeast two-hybridization (Y2H) assay, we constructed the bait plasmid pGBKT7-α chain FnⅢ and pGBKT7-β chain FnⅢ as baits, respectively. For the prey plasmid construction, we cloned the full-length cDNA of *Sb*Fn into the pGADT7 vector. Subsequently, we used the Matchmaker^®^ Gold Yeast Two-Hybrid System (TaKaRa, Mountain View, CA, USA) to execute a Y2H screening. In brief, the bait plasmid (FnⅢ-α/β-BD) and prey plasmid (Fn-AD) were transferred into yeast strains Y2HGold and Y187, respectively. Meanwhile, the blank pGBKT7 (CK-BD) and pGADT7 (CK-AD) vectors were treated identically as negative controls to exclude false positives. The experimental groups consisted of the Y2Hgold yeast strain transfected with FnIII-α/β-BD hybridized with the Y187 yeast strain transfected with Fn-AD, respectively. The Y2Hgold yeast strain transfected with CK-BD was hybridized with the Y187 yeast strain transfected with CK-AD to serve as a negative control. For the self-activation assay, the Y2Hgold yeast strain transfected with FnIII-α/β-BD was hybridized with the Y187 yeast strain transfected with CK-AD and the Y187 yeast strain transfected with Fn-AD was hybridized with the Y2Hgold yeast strain transfected with CK-BD. After hybridization, the cultured yeasts were diluted 10, 100, and 1000-fold with sterile water and inoculated on duplex dropout medium (SD-Leu-Trp) and quadruple dropout medium supplemented with X-a-Gal and Aba (SD-Leu-Trp-His-Ade/X-a-gal/Aba). Successful interactions were dependent on the presence of blue cells on the medium.

### 4.9. Statistical Analysis

All the experiments were repeated independently three times, and the data are presented as mean ± SD. Statistical analysis of the data was carried out using the Statistical Package for Social Sciences (SPSS) 21.0. The differences between the groups were assessed using one-way ANOVA. The differences are considered significant at * *p* < 0.05, ** *p* < 0.01.

## Figures and Tables

**Table 1:** The sequences utilized in this study.

Primer Name	Primer Sequence (5′-3′)
Gene clone primer	
*Sb*Fn-F	CTCCGAAACTCCGCCATATTCTCAC
*Sb*Fn-R	CATGGAATTTATTTAGAAAAAGGACTG
*Sb*IGF-1R-F	ATGACACCACAAAGTGTGGCATCAG
*Sb*IGF-1R-R	TCAGCATGATGTCATCCTCATGTGT
Real-time quantitative PCR primers	
q*Sb*IGF-1R-F	GAAGATCACTGCCGAAGAAGGA
q*Sb*IGF-1R-R	CGTGGAATGCATACAAGAGGTTTC
q*Sb*RL15-F	AGACCAGACAAAGCCAGAAGAC
q*Sb*RL15-R	GCTGAAGTAAGTCCACGCATT
Vector construction primers	(The underline means restriction enzyme site)
*Sb*Fn-BamH I	CGCGGATCCATGGCTCAAACACAACCAAG
*Sb*Fn-Xho I	CCGCTCGAGTATATGTAGATAAAAGCCTAA
*Sb*IGF-1R-FNIII-BamH I	CGCGGATCCTATGTAACTCAACTCAACCTGACTA
*Sb*IGF-1R-FNIII-Xho I	CCGCTCGAGTCTAAAAATGTTTATATGATGTCCATTCT
FnⅢ-α-BD-BamH I	CGCGGATCCGTTATGTAACTCAACTCAACCTGACTA
FnⅢ-α-BD-Not I	AAGGAAAAAAGCGGCCGCTTTACGCCAATACACTTCGTAATGA
FnⅢ-β-BD-BamH I	CGCGGATCCGTGCAGTCGATGTGAATGAAACTGAGG
FnⅢ-β-BD-Not I	AAGGAAAAAAGCGGCCGCAAAATGTTTATATGATGTCCATTCT
*Sb*Fn-AD-EcoR I	CCGGAATTCGCTCAAACACAACCAAGACAAAACT
*Sb*Fn-AD-BamH I	CGCGGATCCCACTGCTCATGGTTTCCTTGTCATAC

## Data Availability

Not applicable.

## Supplementary Materials

The following supporting information can be downloaded at: https://www.mdpi.com/article/10.3390/ijms24076175/s1.
