# Supplementary material for: A Putative Receptor for Ferritin in Mollusks: Characterization of the Insulin-like Growth Factor Type 1 Receptor
Source: Int J Mol Sci. 2023 Mar 24;24(7):6175. doi: 10.3390/ijms24076175 (PMC10094261; doi:10.3390/ijms24076175)
Supplement: Supplementary file 1 [file ijms-24-06175-s001.zip › ijms-2202723-supplementary.pdf]

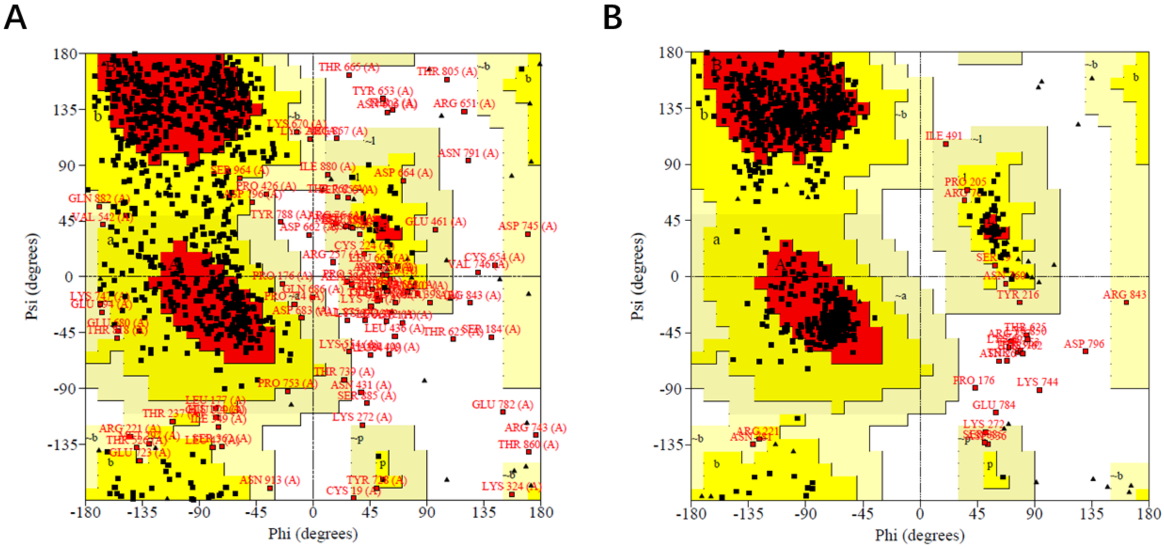

Figure S1. Evaluation of a three-dimensional model of *Sb*IGF-1R. Ramachandran diagrams were used to analyze the probability of amino acids to form secondary structures based on their dihedral angles, i.e., ϕ and Ψ (allowed and disallowed), and to assess the quality of the tertiary structure model by the percentage of residues in the favored, allowed, and outlier regions. The Ramachandran plots show that, in the original model, 58.1%, 32.1%, and 6.4% of the residuals were in the favored, allowed, and outlier regions, respectively (**A**), while in the refined model, 84.7%, 12.6%, and 1.3% of the residuals were in the favored, allowed, and outlier regions, respectively (**B**).
